# Supplementary material for: Staphylococcus aureus binding to Seraph® 100 Microbind® Affinity Filter: Effects of surface protein expression and treatment duration
Source: PLoS One. 2023 Mar 17;18(3):e0283304. doi: 10.1371/journal.pone.0283304 (PMC10022791; doi:10.1371/journal.pone.0283304)
Supplement: Attachment — Submitted filename: PONE-D-22-34334_reviewed.pdf [file pone.0283304.s004.pdf]

PLOS ONE

Staphylococcus aureus binding to Seraph® 100 Microbind® Affinity Filter: Effects of surface protein expression and treatment duration

--Manuscript Draft--

**Manuscript Number:**	PONE-D-22-34334
**Article Type:**	Research Article
**Full Title:**	Staphylococcus aureus binding to Seraph® 100 Microbind® Affinity Filter: Effects of surface protein expression and treatment duration
**Short Title:**	S. aureus binding to Seraph Affinity Filter
**Corresponding Author:**	Susanne Engelmann<br>Technical University Braunschweig<br>Braunschweig, GERMANY
**Keywords:**	Staphylococcus aureus; heparin binding; multiresistent bacteria; blood stream infections; Extracorporeal blood purification systems
**Abstract:**	Extracorporeal blood purification systems represent a promising alternative for treatment of blood stream infections with multiresistent bacteria. The aim of this study was to analyse the binding activity of S. aureus to Seraph affinity filters based on heparin coated beads and to identify effectors influencing this binding activity.To test the binding activity, we used gfp expressing S. aureus Newman strains (wild type and regulatory mutants) either inoculated in 0.9% NaCl or in blood plasma and determined the number of unbound bacteria via FACS analyses after passing Seraph affinity filters. Our main findings are: i) S. aureus binding to heparinized beads is impaired by components present in human blood plasma, ii) differential expression of surface-associated proteins does not significantly influence the binding activity of S. aureus to Seraph, and iii) final reduction rates for S. aureus in human blood plasma of at least 80% are reached after a treatment for four hours and are donor dependent.
**Order of Authors:**	Malin-Theres Seffer
	Martin Weinert
	Gabriella Molinari
	Manfred Rohde
	Lothar Gröbe
	Jan T Kielstein
	Susanne Engelmann
**Additional Information:**	
**Question**	**Response**
**Financial Disclosure**<br><br>Enter a financial disclosure statement that describes the sources of funding for the work included in this submission. Review the submission guidelines for detailed requirements. View published research articles from *PLOS ONE* for specific examples.<br><br>This statement is required for submission and **will appear in the published article** if the submission is accepted. Please make	The author(s) received no specific funding for this work.

sure it is accurate.<br><br>**Unfunded studies**<br>Enter: *The author(s) received no specific funding for this work.*<br><br>**Funded studies**<br>Enter a statement with the following details:<br>• Initials of the authors who received each award<br>• Grant numbers awarded to each author<br>• The full name of each funder<br>• URL of each funder website<br>• Did the sponsors or funders play any role in the study design, data collection and analysis, decision to publish, or preparation of the manuscript?<br>• **NO** - Include this sentence at the end of your statement: *The funders had no role in study design, data collection and analysis, decision to publish, or preparation of the manuscript.*<br>• **YES** - Specify the role(s) played.<br><br>* typeset	
**Competing Interests**<br><br>Use the instructions below to enter a competing interest statement for this submission. On behalf of all authors, disclose any competing interests that could be perceived to bias this work—acknowledging all financial support and any other relevant financial or non-financial competing interests.<br><br>This statement is required for submission and **will appear in the published article** if the submission is accepted. Please make sure it is accurate and that any funding sources listed in your Funding Information later in the submission form are also declared in your Financial Disclosure statement.<br><br>View published research articles from *PLOS ONE* for specific examples.	The authors have declared that no competing interests exist.

**NO authors have competing interests**<br><br>Enter: *The authors have declared that no competing interests exist.*<br><br>**Authors with competing interests**<br><br>Enter competing interest details beginning with this statement:<br><br>*I have read the journal's policy and the authors of this manuscript have the following competing interests: [insert competing interests here]*<br><br>* typeset	
**Ethics Statement**<br><br>Enter an ethics statement for this submission. This statement is required if the study involved:<br><br>• Human participants<br>• Human specimens or tissue<br>• Vertebrate animals or cephalopods<br>• Vertebrate embryos or tissues<br>• Field research<br><br>Write "N/A" if the submission does not require an ethics statement.<br><br>General guidance is provided below. Consult the submission guidelines for detailed instructions. **Make sure that all information entered here is included in the Methods section of the manuscript.**	Ethics committee Hannover Medical School<br>8634_BO-K_2019<br>written consent

Powered by Editorial Manager® and ProduXion Manager® from Aries Systems Corporation

**Format for specific study types**<br><br>**Human Subject Research (involving human participants and/or tissue)**<br>• Give the name of the institutional review board or ethics committee that approved the study<br>• Include the approval number and/or a statement indicating approval of this research<br>• Indicate the form of consent obtained (written/oral) or the reason that consent was not obtained (e.g. the data were analyzed anonymously)<br><br>**Animal Research (involving vertebrate animals, embryos or tissues)**<br>• Provide the name of the Institutional Animal Care and Use Committee (IACUC) or other relevant ethics board that reviewed the study protocol, and indicate whether they approved this research or granted a formal waiver of ethical approval<br>• Include an approval number if one was obtained<br>• If the study involved *non-human primates*, add *additional details* about animal welfare and steps taken to ameliorate suffering<br>• If anesthesia, euthanasia, or any kind of animal sacrifice is part of the study, include briefly which substances and/or methods were applied<br><br>**Field Research**<br><br>Include the following details if this study involves the collection of plant, animal, or other materials from a natural setting:<br>• Field permit number<br>• Name of the institution or relevant body that granted permission	
**Data Availability**<br><br>Authors are required to make all data underlying the findings described fully available, without restriction, and from the time of publication. PLOS allows rare exceptions to address legal and ethical concerns. See the PLOS Data Policy and FAQ for detailed information.	Yes - all data are fully available without restriction

A Data Availability Statement describing where the data can be found is required at submission. Your answers to this question constitute the Data Availability Statement and **will be published in the article**, if accepted.<br><br>**Important:** Stating 'data available on request from the author' is not sufficient. If your data are only available upon request, select 'No' for the first question and explain your exceptional situation in the text box.<br><br>Do the authors confirm that all data underlying the findings described in their manuscript are fully available without restriction?	
**Describe where the data may be found in full sentences. If you are copying our sample text, replace any instances of XXX with the appropriate details.**<br><br>• If the data are **held or will be held in a public repository**, include URLs, accession numbers or DOIs. If this information will only be available after acceptance, indicate this by ticking the box below. For example: *All XXX files are available from the XXX database (accession number(s) XXX, XXX.*).<br>• If the data are all contained **within the manuscript and/or Supporting Information files**, enter the following: *All relevant data are within the manuscript and its Supporting Information files.*<br>• If neither of these applies but you are able to provide **details of access elsewhere**, with or without limitations, please do so. For example:<br><br>*Data cannot be shared publicly because of [XXX]. Data are available from the XXX Institutional Data Access / Ethics Committee (contact via XXX) for researchers who meet the criteria for access to confidential data.*<br><br>*The data underlying the results presented in the study are available from (include the name of the third party*	All relevant data are within the manuscript and its Supporting Information files.

and contact information or URL).<br>• This text is appropriate if the data are owned by a third party and authors do not have permission to share the data.<br><br>* typeset	
Additional data availability information:	

Manuscript
Click here to access/download;Manuscript;Seraph_Paper_221214_text.docx

Staphylococcus aureus binding to Seraph® 100 Microbind® Affinity Filter: Effects of surface protein expression and treatment duration

Malin-Theres Seffer[1,2], Martin Weinert[1,3], Gabriella Molinari[4], Manfred Rohde[4], Lothar Gröbe[5], Jan T Kielstein*[2] and Susanne Engelmann*[1,3]

[1]Helmholtz Centre for Infection Research, Microbial Proteomics, Braunschweig, Germany

[2]Medical Clinic V, Nephrology, Rheumatology, Blood Purification, Academic Teaching Hospital Braunschweig, Germany

[3]Technische Universität Braunschweig, Institute for Microbiology, Braunschweig, Germany

[4]Helmholtz Centre for Infection Research, Central Facility of Microscopy, Braunschweig Germany

[5]Helmholtz Centre for Infection Research, Experimental Immunology, Braunschweig, Germany

*both authors contributed equally and should be considered last authors

Corresponding author:

Susanne Engelmann

Microbial Proteomics, Helmholtz Centre for Infection Research

Inhoffenstr. 7, 38124 Braunschweig, GERMANY

Susanne.Engelmann@helmholtz-hzi.de

Phone: +49-531-6181-3041

Running title: *S. aureus* binding to Seraph Affinity Filter

Abstract

Extracorporeal blood purification systems represent a promising alternative for treatment of blood stream infections with multiresistent bacteria. The aim of this study was to analyse the binding activity of *S. aureus* to Seraph affinity filters based on heparin coated beads and to identify effectors influencing this binding activity.To test the binding activity, we used *gfp* expressing *S. aureus* Newman strains (wild type and regulatory mutants) either inoculated in 0.9% NaCl or in blood plasma and determined the number of unbound bacteria via FACS analyses after passing Seraph affinity filters. Our main findings are: i) *S. aureus* binding to heparinized beads is impaired by components present in human blood plasma, ii) differential expression of surface-associated proteins does not significantly influence the binding activity of *S. aureus* to Seraph, and iii) final reduction rates for *S. aureus* in human blood plasma of at least 80% are reached after a treatment for four hours and are donor dependent.

Introduction

Sepsis is a serious worldwide health threat and affects annually 30 to 50 million people worldwide. Despite international guidelines for treatment of sepsis, the mortality rate for severe sepsis and septic shock remains high with 43.6% and 58.8%, respectively (1, 2). Hence, the WHO ranks sepsis a “key issue for global health” (Global report WHO, https://www.who.int/activities/improving-the-prevention-diagnosis-and-clinical-management-of-sepsis). Bacterial infections play an important role as one of the triggering events that result in an inadequate response of the immune system, which may lead to life threatening organ dysfunctions. Increasing antibiotic resistance in bacteria further complicates treatment of these infections.

Staphylococcus aureus is one of the most frequent and dangerous pathogens that, in addition to mild skin and soft tissue infections, can cause systemic infections as pneumonia, endocarditis or sepsis (3, 4). Patients after surgery or those receiving continuous ambulatory peritoneal dialysis or hemodialysis have a higher risk to get infected with this pathogen. Existing *S. aureus* colonization of the nasopharynx is the main source of subsequent infections (5, 6). Very frequently, these infections are associated with multi-resistant *S. aureus* isolates that are extremely difficult to treat. To illustrate the seriousness of the situation, in 2017, an expert panel of the WHO identified *S. aureus* as a pathogen with high priority for research, discovery, and development of new antibiotics (7).

Antibiotics, once our most effective weapon against bacterial infections, are becoming increasingly less potent and the introduction of novel antibiotics is a lengthy and very expensive process. There has thus been an urgent need for alternative treatment options already for a long time. Physical reduction of bacteria using an extracorporeal device that purifies circulating body fluids represents one attractive alternative (8-12). In 2019, the first biomimetic haemoperfusion device able to reduce the number of pathogens in the blood, the Seraph® 100 Microbind®

Affinity Filter (ExThera Medical, Martinez, CA, USA) was licensed by the European Union. It uses covalently endpoint attached heparin-coated ultrahigh-molecular weight polyethylene to remove bacteria and viruses from blood by exploiting their capacity to interact with heparin (12). Heparin is a well-known anticoagulant drug and mimics heparan sulfate that is normally expressed on cell surfaces (13, 14). Many bacteria, viruses, and toxins adhere to human cells via heparan sulfate mainly mediated by charge or electrostatic interaction (13, 15, 16). Binding to heparin-coated beads has been shown not only for bacteria that are known to express heparin binding proteins such as *S. aureus* and *Staphylococcus epidermidis*, but also for Gram-negative bacteria like *Klebsiella pneumoniae* or *Escherichia coli* (12, 17, 18). Recent studies suggest that also viruses or specific components of viruses as for instance the spike protein and the N-protein of SARS-CoV-2 can be eliminated from blood by binding to the Seraph® 100 Microbind® Affinity Filter (19-21).

Here, we report the impact of surface protein expression in *S. aureus* on its binding activity to heparin coated beads using miniaturized Seraph adsorbers. Moreover, we analysed the influence of blood plasma from different donors and treatment duration on the ability of Seraph® 100 Microbind® Affinity Filter to reduce *S. aureus* from circulating blood plasma.

Material and methods

Bacterial strains, plasmids and human blood plasma

S. aureus strains and plasmids used in this study are listed in **Tab. 1**. The *sarA* deletion mutant of strain Newman (Newman Δ*sarA*) was constructed by transduction of the *sarA*::Tn9J7LTV1 mutation from strain R (22) to strain Newman (23) using phage Φ80 (24). To generate *gfp* expressing *S. aureus* strains, the *gfp* containing plasmid pCtufgfp (25) was transferred into *S. aureus* Newman and its isogenic mutants by phage transduction with bacteriophage Φ80

(24). For selection of plasmid containing strains, the medium was supplemented with chloramphenicol (10 µg/mL).

Plasma samples were obtained from patients after a routine therapeutic plasma exchange and stored up to 48 hours at 4°C. Written informed consent was obtained from the voluntary plasma donors. The study protocol was approved by the Hannover Medical School Ethics Committee and was performed in accordance with the Declaration of Helsinki and German federal guidelines. For micro column experiments, 10 mL aliquots of human blood plasma from one of the donors were stored at -20°C for at least 10 days.

Table 1: Bacterial strains used in this study

Strains	Genotype or characteristics	Reference
S. aureus Newman	Reference strain	(23)
S. aureus R	RN6390 *sar::Tn9J7LTV1*	(22)
S. aureus Newman Δ*lgt*	*lgt*::*ermB*, isogenic to strain Newman	(26)
S. aureus Newman Δ*sarA*	*sarA*::Tn9J7LTV1, isogenic to strain Newman	This study
S. aureus Newman Δ*sigB*	*sigB*::*ermB*, isogenic to strain Newman	(27)
S. aureus Newman Δ*saeS*	*saeS*::Tn917, isogenic to strain Newman	(28)
S. aureus Newman *gfp*$^+$	*S. aureus* Newman containing pCtufgfp	This study
S. aureus Newman Δ*lgt gfp*$^+$	*S. aureus* Newman Δ*lgt* containing pCtufgfp	This study
S. aureus Newman Δ*sarA gfp*$^+$	*S. aureus* Newman Δ*sarA* containing pCtufgfp	This study
S. aureus Newman Δ*sigB gfp*$^+$	*S. aureus* Newman Δ*sigB* containing pCtufgfp	This study
S. aureus Newman Δ*saeS gfp*$^+$	*S. aureus* Newman Δ*saeS* containing pCtufgfp	This study

S. aureus binding experiments with miniaturized Seraph adsorbers (micro columns)

S. aureus strains were cultivated in chemically defined medium (CDM) (29, 30) at 37°C and 120 revolutions per minute (rpm) to an optical density at 500 nm (OD_{500}) of 0.5. Miniaturized

Seraph adsorbers (micro columns), which are hand-packed with media and sterilized by ethylenoxide (ExThera Medical, Martinez, CA, USA), with a total volume of 2.5 mL (for bacterial binding experiments with different mutants in 0.9% NaCl) or 5 mL (for bacterial binding comparing the impact of 0.9% NaCl or human blood plasma as bacterial solution) were pretreated with 5 mL (10 mL) 0.9% NaCl solution before loading with bacterial solutions containing ~ $3*10^5$ bacteria/mL either in 0.9% NaCl (=2.5 mL/5 mL) or in human blood plasma (=5 mL) from one single donor. Subsequently, micro columns were washed twice with 2.5 mL (5 mL) 0.9% NaCl to completely remove unbound bacteria from the column. Free flows of the loading step (Eluate 1) and of each washing step (Eluate 2 and 3) were collected and analysed by FACS. Non-passed bacterial solutions either in 0.9% NaCl or in blood plasma served as control. A schematic overview of the experimental workflow is presented in **Fig. 1**.

Figure 1 Schematic overview of the experimental setup using microcolumns. After pretreatment with 0.9% NaCl solution, microcolumns were loaded with bacterial solutions containing ~ $3*10^5$ bacteria/ml and washed two times with 0.9% NaCl solution. Eluate 1, 2 and 3 and the control samples were analysed by FACS.

S. aureus **binding experiments with Seraph® 100 Microbind® Affinity blood adsorber**

To study long-term treatment under more relevant clinical conditions, Seraph® 100 Microbind® Affinity blood adsorber (ExThera Medical, Martinez, CA, USA), used for clinical applications, was attached to an AFERsmart hemofiltration device (Medica S.a.P., Italia) and a tubing system (Meise Medizintechnik GmbH, Schalksmühle, Germany) thereby establishing a closed circuit of human plasma with constant flow rates of 60 mL/min (31). First, the Seraph® 100 Microbind® Affinity blood adsorber was pretreated with 500 mL 0.9% NaCl solution. Afterwards, a plasma bag containing 1000 mL blood plasma was inoculated with *S. aureus*

(cultivated in synthetic medium to an OD_{500} of 0.5) to a final concentration to $3x10^5$ bacteria/mL and connected to the blood adsorber. After defined time periods, samples were taken and stored on ice until FACS analysis. A schematic overview of the experimental workflow is provided in **Fig. 2**. Each replicate has been performed with blood plasma from a separate donor (n=4).

Figure 2 Schematic overview of the experimental setup using the Seraph® 100 Microbind® Affinity blood adsorber. After pretreatment of the Seraph® 100 Microbind® Affinity blood adsorber with 500 ml 0.9% NaCl solution**,** 1000 ml Plasma were spiked with ~ $3*10^5$ bacteria/ml and circulated through the Seraph cartridge by a roller pump. Plasma samples were obtained at different time points and analysed by FACS.

Bacteria cell counting and enumeration assay

To determine numbers of *gfp*-expressing bacteria in a defined sample volume, fluorescence activated cell sorting (FACS) analyses were performed using an LSR II flow cytometer (Becton-Dickinson™). Samples were homogenized by shaking and diluted in 0.9% NaCl (1:2 for NaCl samples and 1:5 for plasma samples). A calibrated flow cytometry cell counting bead solution (6 µm diameter) (ThermoFisher Scientific, Waltham, MA, USA, Invitrogen) was homogenized by shaking and treatment in an ultrasonic bath (5 min at room temperature, 80 Hz). 5 µL aliquotes of the counting bead solution were added to 1000 µL of each FACS sample. Immediately before FACS analyses, samples were shaken for >5 s.

The cytometer was set up for detecting forward scatter (FSC; bandpass filter 488+/-5 nm), side scatter (SSC; bandpass filter 488+/-5 nm) and green fluorescence (excitation 488 nm, bandpass

filter 530+/-15 nm), all in logarithmic scale. SSC served as the trigger signal. Beads were gated by forward scatter (FSC) and side scatter (SSC). GFP expressing bacteria were gated by green fluorescence and SSC. 10.000 bead events were recorded and served as reference for sample volume. The respective number of detected bacteria was used to calculate the bacterial cell concentration in the samples.

Data analyses and statistics

The bacterial cell concentration based on detected GFP signals was calculated by using the following formula:

$$\boldsymbol{C = \frac{N_{GFP} * f_{elu}}{N_{beads} * V_b}}$$

(N_{GFP}= Number of detected GFP-signals; f_{elu}= eluting factor; N_{beads}=number of detected bead events; V_b = liquid volume).

The total reduction rate (RR_{total}) of bacteria using the miniaturized Seraph adsorbers was calculated as follows:

$$\boldsymbol{RR_{total} = 1 - \frac{N_1 + N_2 + N_3}{N_c}}$$

(N_c = bacterial cell number of the control solution (**Fig. 1**); $N_{1\ to\ 3}$ = bacterial cell number of eluates 1 to 3 (**Fig. 1**)). To test whether differences in binding activity exist between different *S. aureus* strains, t-test analyses were performed.

Field Emission Scanning Electron Microscopy (FESEM)#

S. aureus was cultivated in synthetic medium to an optical density of 2.4. 500 µL culture volume (~ $1*10^8$ bacteria) were added to 25 mg heparin-coated beads and fixed with 2% glutaraldehyde in medium. Beads were placed onto filter paper in a funnel and washed with TE buffer (20 mM TRIS, 1 mM EDTA, pH 6.9). Dehydration was achieved with a graded series of ethanol followed by a gradient of tetramethylsilan (TMS) by gently adding the different liquids dropwise over the filter paper. After air-drying, samples were mounted onto aluminium stubs with adhesive tape and covered with a gold/palladium film by sputter coating (SCD 500 Bal-Tec, Liechtenstein) before examination in a field emission scanning electron microscope Zeiss Merlin (Zeiss, Oberkochen). Images were taken with the SEM software version 5.05 at an acceleration voltage of 5 kV with the Inlens SE-detector and HESE2 SE-detector in a 75:25 ratio.

Results

Binding activity of *S. aureus* Newman to Seraph micro columns

To determine the binding activity of *S. aureus* to the heparin-coated pathogen adsorber, Seraph micro columns were loaded with a defined number of bacterial cells. For these experiments, *S. aureus* Newman was used as a reference strain and suspended either in 0.9% NaCl solution or human blood plasma. After passing the columns, the eluate was collected (eluate 1). To completely remove all unbound bacteria, the columns were washed twice with 0.9% NaCl (eluate 2 and 3). Bacterial cells in all three eluates were enumerated by FACS and the number of bound bacteria was calculated (**Fig. 1**). Accordingly, of $2.7*10^6$ *S. aureus* Newman cells suspended in 0.9% NaCl $1.13*10^6$ cells were bound to Seraph micro columns (5 mL), which corresponds to a reduction rate of 0.42 (**Tab. 2**). *S. aureus* binding to heparin-coated beads was visualized by electron microscopy (**Fig. 3**). When using *S. aureus* Newman suspended in human blood plasma, *S. aureus* binding activity to the adsorber was significantly lower. The number

of bound bacteria was about 2.77*10^5 (**Tab. 2**). The resulting reduction rate was not more than 0.10. It appeared that proteins or other components present in plasma interfered with *S. aureus* binding to heparinized beads. They may directly bind to heparan sulfate and thus compete with *S. aureus* for binding to these molecules or they target *S. aureus* surface and sterically hinder access of surface expressed *S. aureus* heparin binding proteins to heparan sulfate on the beads.

Figure 3 Scanning electron microscopy of *S. aureus* Newman attached to heparin-coated beads. Approximately 1.5*10^8 bacteria were added to 25 mg of the Seraph's material and fixed with 2% Glutardaldehyd

Table 2: Binding activity of *S. aureus* Newman to Seraph microcolumns

	original bacterial solution		bound bacteria		reduction rate
	bacterial cell number	Standard deviation	bacterial cell number	Standard deviation	
in 0.9% NaCl	2.70*10^6	27%	1.13*10^6	8%	**42%**
in human plasma	2.85*10^6	2%	2.77*10^5	7%	**10%**

Impact of surface protein expression in *S. aureus* on its binding activity to Seraph

The composition of the surface proteome is highly variable among different *S. aureus* isolates and might be relevant for its binding activity to the adsorber (32-35). In order to prove this, the binding activity of *S. aureus* Newman wild type was compared to that of isogenic mutants with strong effects on surface protein expression. Among them are mutants in *sarA, saeS, lgt* and *sigB*. While the loss of the prolipoprotein diacylglyceryl transferase Lgt completely prevents the attachment of lipoproteins to the cell surface in *S. aureus* (26), the deficiency of regulatory proteins such as SarA, SigB and SaeS impacts the expression of specific sets of genes coding for surface associate proteins in this pathogen (33, 34, 36-40), For these experiments,

miniaturized Seraph adsorbers (2.5 mL) were loaded with 3.8 to 7.9*10^5 *S. aureus* wild type or mutant cells per mL suspended in 0.9% NaCL and the number of bound bacteria was determined as aforementioned. Reduction rates for the Δ*lgt*, Δ*sigB*, Δ*sarA*, and Δ*saeS* mutants were 0.54, 0.55, 0.70 and 0.63 and do not differ significantly among each other and to that of the wild type (0.61) (**Tab. 3**). These data suggest that differences in protein composition on the cell surface of *S. aureus* induced by different activities of essential virulence regulators such as SarA, SigB and Sae or by a defect in the attachment of lipoproteins to the bacterial surface had only minor influence on *S. aureus* binding to heparinized beads.

Table 3: Binding activity of different *S. aureus* mutants to Seraph microcolumns

	original bacterial solution		bound bacteria		reduction rate
	total bacterial cell number	Standard deviation	total bacterial cell number	Standard deviation	
wild type	1.93*10^6	6%	1.17*10^6	0.27%	**60.65%**
sigB mutant	1.81*10^6	13%	9.97*10^5	0.08%	**55.19%**
saeS mutant	1.79*10^6	7%	1.12*10^6	0.14%	**62.56%**
sarA mutant	9.56*10^5	10%	6.68*10^5	0.14%	**69.85%**
lgt mutant	1.96*10^6	6%	1.06*10^6	0.13%	**54.07%**

S. aureus binding activity to Seraph during a prolonged treatment period

To study *S. aureus* binding activity under conditions mimicking the clinical situation, we used the Seraph® 100 Microbind® Affinity blood adsorber that was connected to a hemofiltration device and a tubing system. In this way, we established a closed circular flow in which *S. aureus* containing human blood plasma samples (1000 mL) passed the adsorber by a constant flow rate (60 mL/min). 1000 ml human blood plasma was initially inoculated with ~ 4.46*10^8 bacteria and samples were taken before and at defined time points (10 min, 30 min, 60 min, 120 min, 240 min, 360 min, 480 min, 600 min, 1080 min) after starting circulation. A continuous

decrease of *gfp* expressing *S. aureus* cells in blood plasma was observed within the first four hours (**Fig. 4**). After two hours, 52 to 64% of *S. aureus* cells initially inoculated in blood plasma samples were attached to the filter. Four hours later, the number of *gfp* expressing *S. aureus* cells did not changed significantly. At this time point, each blood plasma sample has passed the filter almost 22 times. During this procedure, $3.9*10^8$ to $4.5*10^8$ *gfp* expressing *S. aureus* cells were bound to the filter (**Tab. S1**). Final reduction rates after 8 h circulation are donor dependent and varied between 0.8 and 1.0 (**Fig. 4**).

Figure 4 *S. aureus* binding activity to Seraph during a prolonged treatment period. Plasma samples of 1000 mL obtained from four different donors were inoculated with $\sim 4.5*10^8$ bacteria and circulated through the Seraph® 100 Microbind® Affinity blood adsorber. Samples were taken at different time points and analysed by FACS. The number of unbound bacteria were calculated for each sampling point.

Discussion

The Seraph® 100 Microbind® Affinity Blood Filter (ExThera Medical Corp., Martinez, CA, USA) is the first CE-approved extracorporeal filter in use to eliminate bacteria from human blood. Ultra-high molecular weight polyethylene beads with endpoint-attached heparin are utilized to reduce bacterial load in blood. Pathogens bind to heparin via inherent adhesion mechanisms which are not completely understood. The aim of this study was to analyse the binding activity of *S. aureus* to the adsorber and to identify effectors influencing *S. aureus* binding. The novel findings of this study are: i) components present in human blood plasma interfere with *S. aureus* binding to heparinized beads, ii) differential expression of surface-associated proteins did not significantly influence the binding activity of *S. aureus* to Seraph

and iii) final reduction rates of at least 80% for inoculated *S. aureus* in 1000 mL human blood plasma were reached after a treatment for four hours and were donor dependent.

Many bacteria have been shown to exploit heparan sulfate that is expressed on the cell surface of eukaryotic cells as receptor for cell adhesion. However, mechanisms by which bacteria bind to heparan sulfate or heparin are still not adequately understood. Multiple surface associated heparin binding proteins were identified in *S. aureus* (41, 42). Although Protein A was identified as one of the most abundant heparin binding proteins in *S. aureus* (41), a loss of protein A did not completely abolished heparin binding of *S. aureus* (43). These data clearly suggest that *S. aureus* binding to heparin is a multifactorial process. This is also supported by the fact that although we used several strains that are strongly impaired in surface protein expression, none of these strains showed significantly different heparin binding capabilities. This finding also implies that clinical *S. aureus* isolates with varying surface protein expressions will be removed from blood without significant differences using heparin coated beads. This is supported by a previous study showing that MRSA (n=6) and MSSA (n=1) isolates in whole blood bind to heparin with similar affinity (18).

Heparin has been known for long time to prevent thrombosis and is thus routinely used in central venous catheters. *S. aureus* and coagulase negative isolates incorporate heparin into the biofilm matrix which significantly increases the biofilm capacity (41, 44, 45). This is an undesirable and often problematic side effect and may also play a significant role for treatment success using Seraph. With our approach, we can clearly estimate that replicating *S. aureus* did not increase bacterial load in plasma during a treatment time of up to eight hours. Since *S. aureus* Newman is unable to form a robust biofilm (46), this study does not allow statements related to the question whether *S. aureus* biofilm formation significantly impacts the reduction rate of *S. aureus*. However, in a first clinical trial, we showed for a female chronic hemodialysis patient with persistent *S. aureus* blood infection that at the end of a four hour hemoperfusion

treatment *S. aureus* was completely eliminated (47). It can be assumed that for such relatively short treatment periods biofilm formation of bacteria bound to the beads does not play a serious role.

Extrapolating from our *in vitro* results, life size Seraph® can bind up to $4*10^8$ *S. aureus* cells in human blood plasma. The bacterial load in bacteraemia patients can reach up to $1*10^4$ CFUs/ml (48) which corresponds to a total number $6*10^5$ CFUs in an average adult (6 L blood). Accordingly, the capacity of Seraph® should be sufficient to reduce the number of *S. aureus* from blood to bacterial cell numbers that could be taken care by the immune system. When applying a flow rate of 60 mL/min the total blood volume of an adult patient should have been passed once through the filter after 100 min. Data of the prolonged treatment period experiment revealed that at least $2*10^8$ bacteria were bound to the filter after 120 min treatment duration, which corresponds to a reduction of the bacterial load by 50%. It is therefore tempting to speculate that at least half of the bacteria present in the blood of patients with bacteremia should be attached to the filter after the first passage. After treatment for more than 4 h, the number of unbound *S. aureus* cells in blood plasma was not further diminished suggesting that from this time point no further bacteria were bound to the filter. Using different plasma samples, we observed a great variance in reduction of *S. aureus* possibly due to differences in the compositions of these samples, strongly suggesting patient variance concerning treatment efficiency. However, the impact of blood cells on *S. aureus* binding to Seraph has been neglected in our studies. We have seen that binding activity of *S. aureus* to heparin coated beads was significantly impaired by components in blood plasma and it is thus very likely that the presence of blood cells will have additional effects. And finally, heart rate, blood pressure and temperature may have an influence as well.

Based on our results, it can be assumed that extracorporeal blood purification treatment based on heparin coated beads is a highly reliable and efficient treatment strategy for *S. aureus* blood

stream infections and represents a highly promising alternative for infections with multiresistent strains. Treatment durations of up to four hours are sufficient to decrease bacterial cell numbers to levels that could be taken care of by the immune system. Initial applications in hospital for persistent *S. aureus* infections are promising and support this hypothesis (47).

Acknowledgements

We thank ExThera Medical Corporation for supplying Seraph adsorber mini columns and Seraph® 100 Microbind® Affinity blood adsorber. We acknowledge Julius Schmidt (Hannover Medical School) for providing plasma samples.

Funding

This work was supported by internal funding.

References

1. Fleischmann C, Thomas-Rueddel DO, Hartmann M, Hartog CS, Welte T, Heublein S, et al. Hospital Incidence and Mortality Rates of Sepsis. Dtsch Arztebl Int. 2016;113(10):159-66.

2. Rhodes A, Evans LE, Alhazzani W, Levy MM, Antonelli M, Ferrer R, et al. Surviving Sepsis Campaign: International Guidelines for Management of Sepsis and Septic Shock: 2016. Intensive Care Med. 2017;43(3):304-77.

3. Tong SY, Davis JS, Eichenberger E, Holland TL, Fowler VG, Jr. *Staphylococcus aureus* infections: epidemiology, pathophysiology, clinical manifestations, and management. Clin Microbiol Rev. 2015;28(3):603-61.

4. Opota O, Croxatto A, Prod'hom G, Greub G. Blood culture-based diagnosis of bacteraemia: state of the art. Clin Microbiol Infect. 2015;21(4):313-22.

5. Pujol M, Pena C, Pallares R, Ariza J, Ayats J, Dominguez MA, et al. Nosocomial *Staphylococcus aureus* bacteremia among nasal carriers of methicillin-resistant and methicillin-susceptible strains. Am J Med. 1996;100(5):509-16.

6. von Eiff C, Becker K, Machka K, Stammer H, Peters G. Nasal carriage as a source of *Staphylococcus aureus* bacteremia. Study Group. N Engl J Med. 2001;344(1):11-6.

7. Tacconelli E, Carrara E, Savoldi A, Harbarth S, Mendelson M, Monnet DL, et al. Discovery, research, and development of new antibiotics: the WHO priority list of antibiotic-resistant bacteria and tuberculosis. Lancet Infect Dis. 2018;18(3):318-27.

8. Didar TF, Cartwright MJ, Rottman M, Graveline AR, Gamini N, Watters AL, et al. Improved treatment of systemic blood infections using antibiotics with extracorporeal opsonin hemoadsorption. Biomaterials. 2015;67:382-92.

9. Liu L, Chen S, Xue Z, Zhang Z, Qiao X, Nie Z, et al. Bacterial capture efficiency in fluid bloodstream improved by bendable nanowires. Nat Commun. 2018;9(1):444.

10. Opal SM. Non-antibiotic treatments for bacterial diseases in an era of progressive antibiotic resistance. Crit Care. 2016;20(1):397.

11. Lee JJ, Jeong KJ, Hashimoto M, Kwon AH, Rwei A, Shankarappa SA, et al. Synthetic ligand-coated magnetic nanoparticles for microfluidic bacterial separation from blood. Nano Lett. 2014;14(1):1-5.

12. Seffer MT, Cottam D, Forni LG, Kielstein JT. Heparin 2.0: A New Approach to the Infection Crisis. Blood Purif. 2021;50(1):28-34.

13. Salmivirta M, Lidholt K, Lindahl U. Heparan sulfate: a piece of information. FASEB J. 1996;10(11):1270-9.

14. Shriver Z, Capila I, Venkataraman G, Sasisekharan R. Heparin and heparan sulfate: analyzing structure and microheterogeneity. Handb Exp Pharmacol. 2012(207):159-76.

15. Cagno V, Tseligka ED, Jones ST, Tapparel C. Heparan Sulfate Proteoglycans and Viral Attachment: True Receptors or Adaptation Bias? Viruses. 2019;11(7).

16. Rostand KS, Esko JD. Microbial adherence to and invasion through proteoglycans. Infect Immun. 1997;65(1):1-8.

17. McCrea K, Ward R, LaRosa SP. Removal of Carbapenem-Resistant Enterobacteriaceae (CRE) from blood by heparin-functional hemoperfusion media. PLoS One. 2014;9(12):e114242.

18. Mattsby-Baltzer I, Bergstrom T, McCrea K, Ward R, Adolfsson L, Larm O. Affinity apheresis for treatment of bacteremia caused by *Staphylococcus aureus* and/or methicillin-resistant S. aureus (MRSA). J Microbiol Biotechnol. 2011;21(6):659-64.

19. Andermatt R, Bloemberg GV, Ganter CC, Mueller NJ, Mueller AMS, Muellhaupt B, et al. Elimination of Herpes Simplex Virus-2 and Epstein-Barr Virus With Seraph 100 Microbind Affinity Blood Filter and Therapeutic Plasma Exchange: An Explorative Study in a Patient With Acute Liver Failure. Crit Care Explor. 2022;4(8):e0745.

20. Olson SW, Oliver JD, Collen J, Bunin J, Gleeson TD, Foster BE, et al. Treatment for Severe Coronavirus Disease 2019 With the Seraph-100 Microbind Affinity Blood Filter. Crit Care Explor. 2020;2(8):e0180.

21. Kielstein JT, Borchina DN, Fuhner T, Hwang S, Mattoon D, Ball AJ. Hemofiltration with the Seraph® 100 Microbind® Affinity filter decreases SARS-CoV-2 nucleocapsid protein in critically ill COVID-19 patients. Crit Care. 2021;25(1):190.

22. Cheung AL, Ying P. Regulation of alpha- and beta-hemolysins by the *sar* locus of *Staphylococcus aureus*. J Bacteriol. 1994;176(3):580-5.

23. Duthie ES, Lorenz LL. Staphylococcal coagulase; mode of action and antigenicity. J Gen Microbiol. 1952;6(1-2):95-107.

24. Rosenblum ED, Tyrone S. Serology, Density, and Morphology of Staphylococcal Phages. J Bacteriol. 1964;88:1737-42.

25. Biswas R, Voggu L, Simon UK, Hentschel P, Thumm G, Götz F. Activity of the major staphylococcal autolysin Atl. FEMS Microbiol Lett. 2006;259(2):260-8.

26. Stoll H, Dengjel J, Nerz C, Götz F. *Staphylococcus aureus* deficient in lipidation of prelipoproteins is attenuated in growth and immune activation. Infect Immun. 2005;73(4):2411-23.

27. Kusch K, Hanke K, Holtfreter S, Schmudde M, Kohler C, Erck C, et al. The influence of SaeRS and sigma(B) on the expression of superantigens in different *Staphylococcus aureus* isolates. Int J Med Microbiol. 2011;301(6):488-99.

28. Goerke C, Fluckiger U, Steinhuber A, Zimmerli W, Wolz C. Impact of the regulatory loci *agr*, *sarA* and *sae* of *Staphylococcus aureus* on the induction of alpha-toxin during device-related infection resolved by direct quantitative transcript analysis. Mol Microbiol. 2001;40(6):1439-47.

29. Engelmann S, Hecker M. Proteomic analysis to investigate regulatory networks in *Staphylococcus aureus*. Methods Mol Biol. 2008;431:25-45.

30. Gertz S, Engelmann S, Schmid R, Ohlsen K, Hacker J, Hecker M. Regulation of sigmaB-dependent transcription of *sigB* and *asp23* in two different *Staphylococcus aureus* strains. Mol Gen Genet. 1999;261(3):558-66.

31. Schmidt JJ, Eden G, Seffer MT, Winkler M, Kielstein JT. In vitro elimination of anti-infective drugs by the Seraph® 100 Microbind® affinity blood filter. Clin Kidney J. 2020;13(3):421-4.

32. Kusch H, Engelmann S. Secrets of the secretome in *Staphylococcus aureus*. Int J Med Microbiol. 2014;304(2):133-41.

33. Rogasch K, Rühmling V, Pané-Farré J, Höper D, Weinberg C, Fuchs S, et al. Influence of the two-component system SaeRS on global gene expression in two different *Staphylococcus aureus* strains. J Bacteriol. 2006;188(22):7742-58.

34. Ziebandt AK, Weber H, Rudolph J, Schmid R, Höper D, Engelmann S, et al. Extracellular proteins of *Staphylococcus aureus* and the role of SarA and sigma B. Proteomics. 2001;1(4):480-93.

35. Ziebandt AK, Kusch H, Degner M, Jaglitz S, Sibbald MJ, Arends JP, et al. Proteomics uncovers extreme heterogeneity in the *Staphylococcus aureus* exoproteome due to genomic plasticity and variant gene regulation. Proteomics. 2010;10(8):1634-44.

36. Bischoff M, Dunman P, Kormanec J, Macapagal D, Murphy E, Mounts W, et al. Microarray-based analysis of the *Staphylococcus aureus* sigmaB regulon. J Bacteriol. 2004;186(13):4085-99.

37. Cassat J, Dunman PM, Murphy E, Projan SJ, Beenken KE, Palm KJ, et al. Transcriptional profiling of a *Staphylococcus aureus* clinical isolate and its isogenic *agr* and *sarA* mutants reveals global differences in comparison to the laboratory strain RN6390. Microbiology (Reading). 2006;152(Pt 10):3075-90.

38. Hempel K, Pané-Farré J, Otto A, Sievers S, Hecker M, Becher D. Quantitative cell surface proteome profiling for SigB-dependent protein expression in the human pathogen *Staphylococcus aureus* via biotinylation approach. J Proteome Res. 2010;9(3):1579-90.
39. Pané-Farré J, Jonas B, Förstner K, Engelmann S, Hecker M. The sigmaB regulon in *Staphylococcus aureus* and its regulation. Int J Med Microbiol. 2006;296(4-5):237-58.
40. Ziebandt AK, Becher D, Ohlsen K, Hacker J, Hecker M, Engelmann S. The influence of *agr* and sigmaB in growth phase dependent regulation of virulence factors in *Staphylococcus aureus*. Proteomics. 2004;4(10):3034-47.
41. Mishra S, Horswill AR. Heparin Mimics Extracellular DNA in Binding to Cell Surface-Localized Proteins and Promoting *Staphylococcus aureus* Biofilm Formation. mSphere. 2017;2(3).
42. Fallgren C, Utt M, Ljungh A. Isolation and characterisation of a 17-kDa staphylococcal heparin-binding protein with broad specificity. J Med Microbiol. 2001;50(6):547-57.
43. Shi H, Tang J, An C, Yang L, Zhou X. Protein A of *Staphylococcus aureus* strain NCTC8325 interacted with heparin. Arch Microbiol. 2021;203(5):2563-73.
44. Ibberson CB, Parlet CP, Kwiecinski J, Crosby HA, Meyerholz DK, Horswill AR. Hyaluronan Modulation Impacts *Staphylococcus aureus* Biofilm Infection. Infect Immun. 2016;84(6):1917-29.
45. Shanks RM, Donegan NP, Graber ML, Buckingham SE, Zegans ME, Cheung AL, et al. Heparin stimulates *Staphylococcus aureus* biofilm formation. Infect Immun. 2005;73(8):4596-606.
46. Cue D, Junecko JM, Lei MG, Blevins JS, Smeltzer MS, Lee CY. SaeRS-dependent inhibition of biofilm formation in *Staphylococcus aureus* Newman. PLoS One. 2015;10(4):e0123027.

47. Seffer MT, Eden G, Engelmann S, Kielstein JT. Elimination of *Staphylococcus aureus* from the bloodstream using a novel biomimetic sorbent haemoperfusion device. BMJ Case Rep. 2020;13(8).

48. Bacconi A, Richmond GS, Baroldi MA, Laffler TG, Blyn LB, Carolan HE, et al. Improved sensitivity for molecular detection of bacterial and *Candida* infections in blood. J Clin Microbiol. 2014;52(9):3164-74.

 Click here to access/download;Figure;Figure 1.pdf

Figure 1

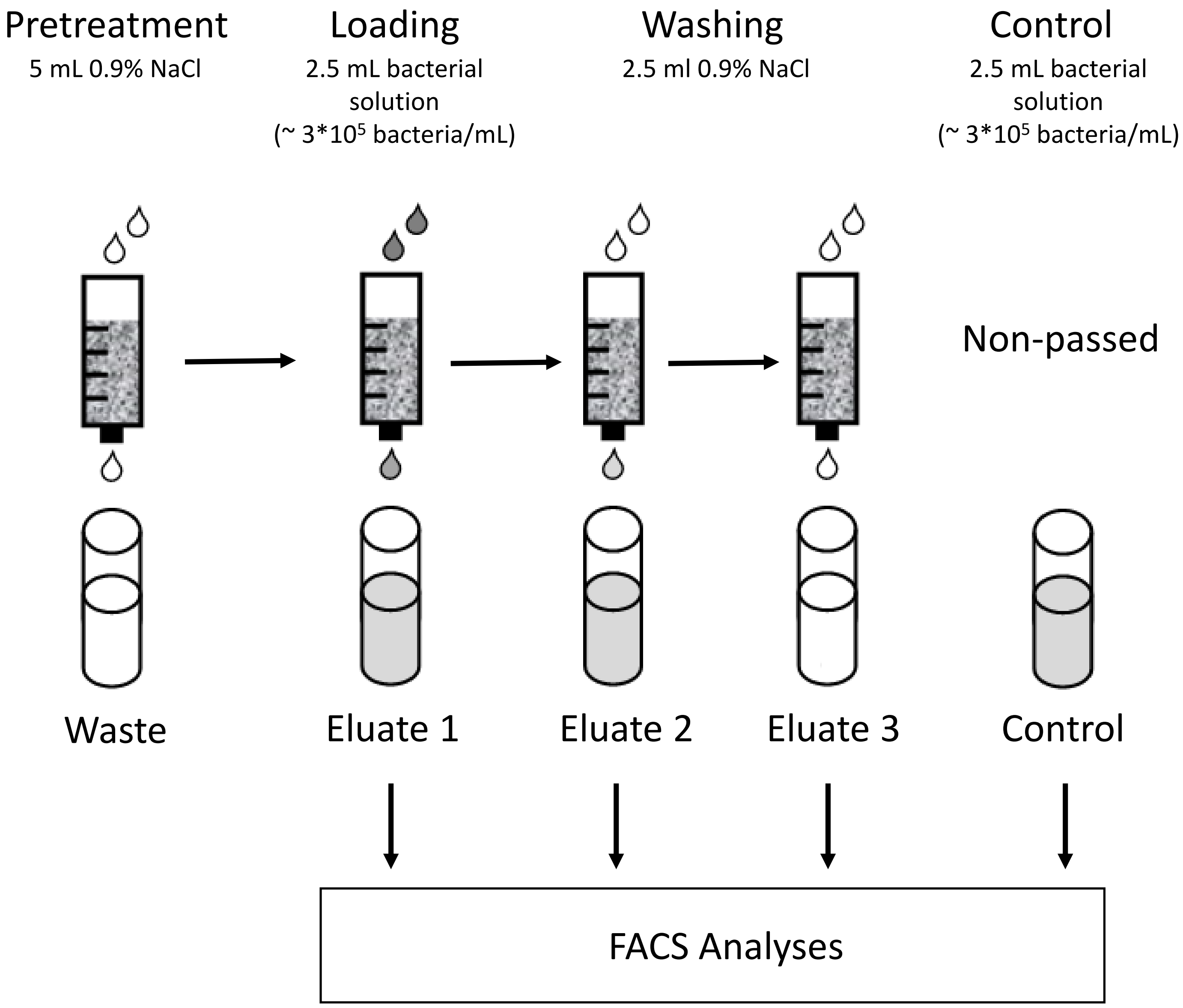

Click here to access/download;Figure;Figure 2.pdf

Figure 2

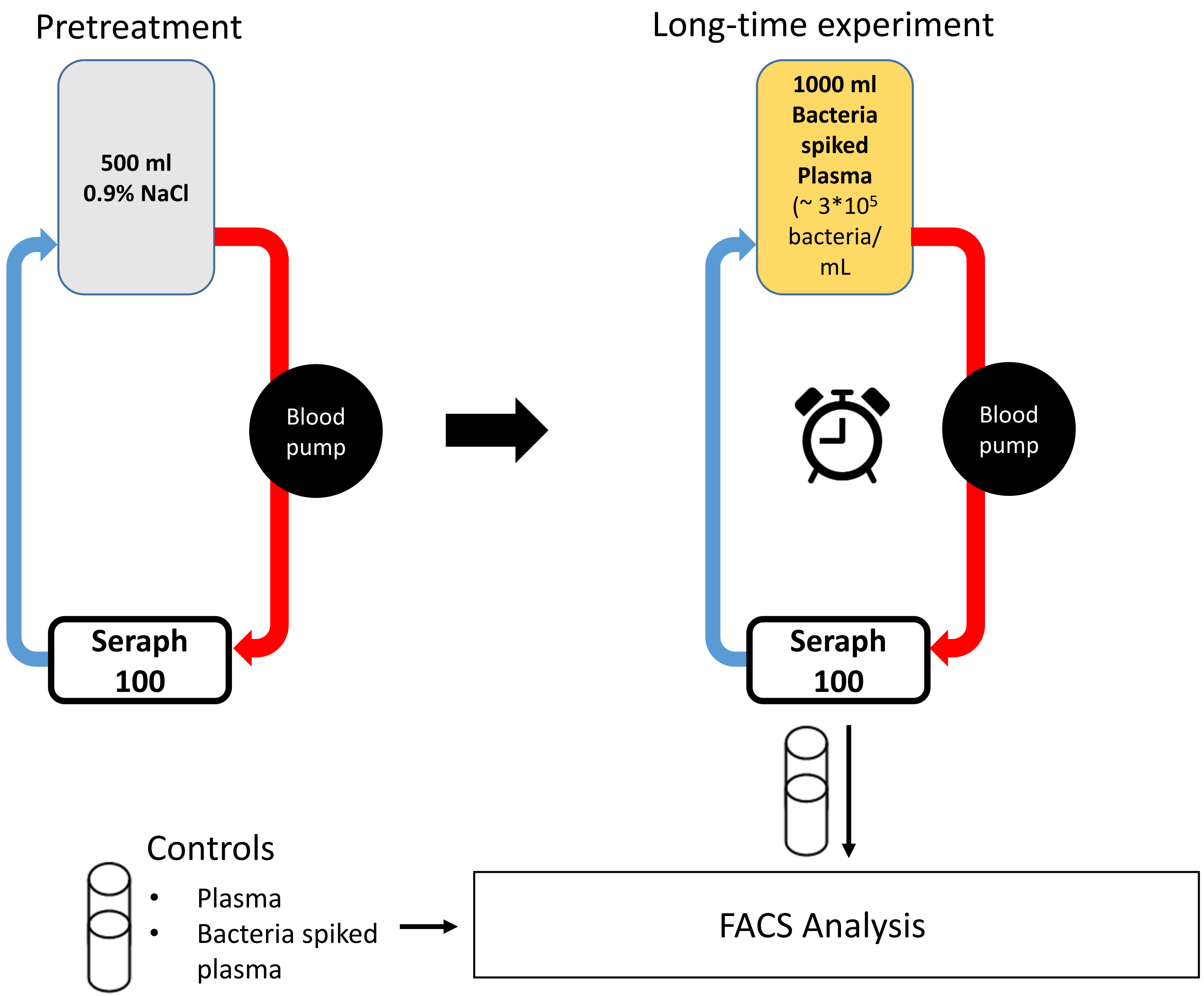

Figure 3

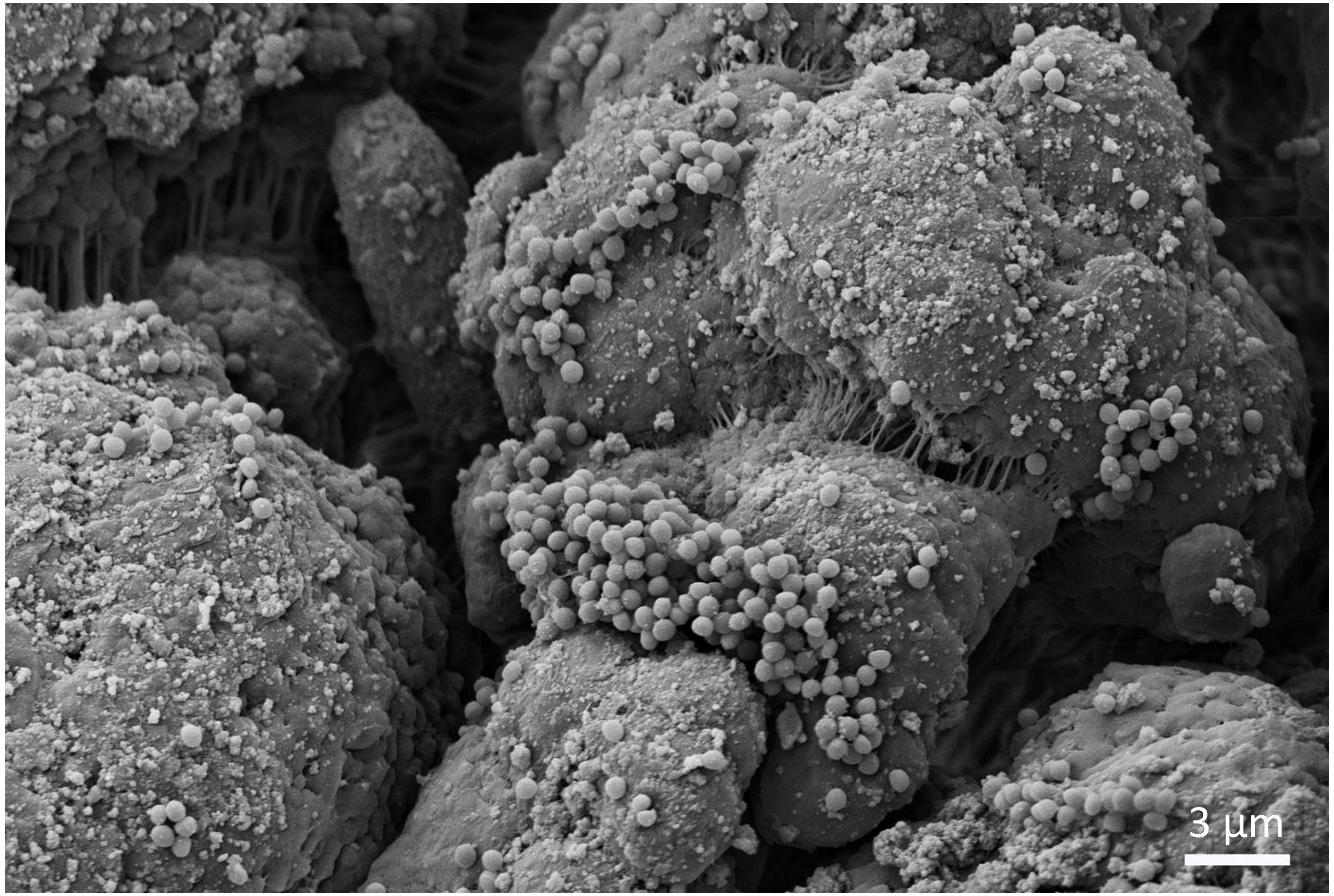

 Click here to access/download;Figure;Figure 4.pdf

Figure 4

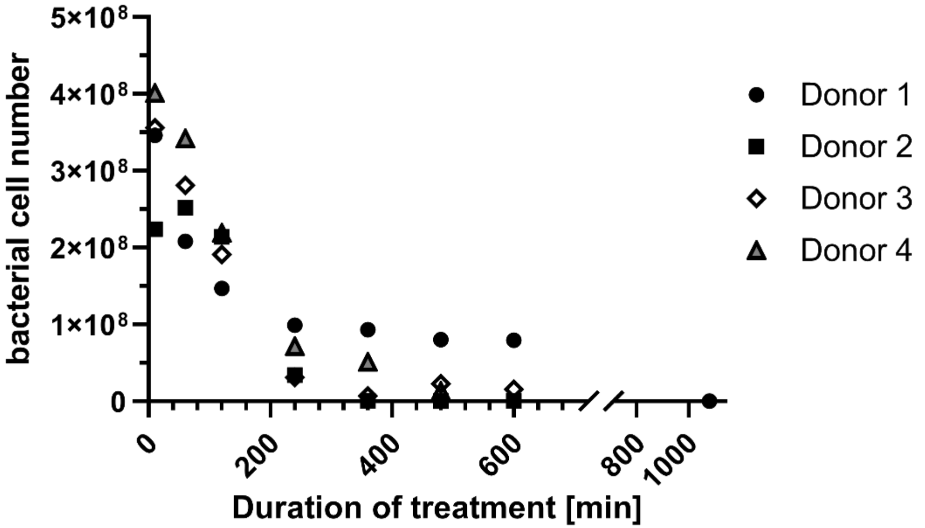

Click here to access/download
Supporting Information
Table_S1.xlsx
